# Antenatal Endotoxin Impairs Lung Mechanics and Increases Sensitivity to Ventilator-Induced Lung Injury in Newborn Rat Pups

**DOI:** 10.3389/fphys.2020.614283

**Published:** 2021-01-13

**Authors:** Erica W. Mandell, Courtney Mattson, Gregory Seedorf, Sharon Ryan, Tania Gonzalez, Alison Wallbank, Elisa M. Bye, Steven H. Abman, Bradford J. Smith

**Affiliations:** ^1^Department of Pediatrics, Pediatric Heart Lung Center, School of Medicine, University of Colorado, Aurora, CO, United States; ^2^Division of Neonatology, Department of Pediatrics, School of Medicine, University of Colorado, Aurora, CO, United States; ^3^Department of Bioengineering, College of Engineering, Design, and Computing, University of Colorado Denver | Anschutz Medical Campus, Aurora, CO, United States; ^4^Division of Pediatric Pulmonary and Sleep Medicine, Department of Pediatrics, School of Medicine, University of Colorado, Aurora, CO, United States

**Keywords:** newborn rat ventilation, lung development, endotoxin, lung structure, ventilator induced lung injury, antenatal exposure

## Abstract

Perinatal inflammation due to chorioamnionitis and ventilator-induced lung injury (VILI) at birth is independent risk factors for the development of bronchopulmonary dysplasia (BPD). We have previously shown that antenatal endotoxin (ETX) causes abnormal lung structure and function in 2-week-old rats, but whether ETX impairs lung mechanics at birth and increases risk for VILI is unknown. Fetal rats were exposed to 10 μg endotoxin or saline *via* intra-amniotic injection. At birth (D0) or 7 days (D7), rats received 90 min of lung protective ventilation [PROTECT group; tidal volume (Vt) = 6 ml/kg with positive end expiratory pressure (PEEP) = 2 cmH_2_O]; P20 ventilation [plateau pressure (Pplat) = 20 cmH_2_O, PEEP = 0]; or P24 ventilation (Pplat = 24 cmH_2_O, PEEP = 0, only applied to D7). Prior to prolonged ventilation at D0, endotoxin-exposed rats had decreased compliance and inspiratory capacity (IC) compared to controls. At D7, endotoxin was associated with reduced compliance. High-pressure ventilation (P20 and P24) tended to increase IC and compliance in all saline-treated groups. Ventilation at D0 with P20 increased IC and compliance when applied to saline-treated but not endotoxin-exposed pups. At D7, P24 ventilation of endotoxin-exposed pups increased elastance, bronchoalveolar lavage protein content, and IL-1b and TEN-C mRNA expression in comparison to the saline group. In summary, antenatal endotoxin exposure alters lung mechanics at birth and 1 week of life and increases susceptibility to VILI as observed in lung mechanics, alveolocapillary barrier injury, and inflammatory mRNA expression. We speculate that antenatal inflammation primes the lung for a more marked VILI response, suggesting an adverse synergistic effect of antenatal and postnatal exposures.

## Introduction

Bronchopulmonary dysplasia (BPD), the chronic lung disease that follows preterm birth, is characterized by persistent respiratory disease and strongly associated with severe lifelong co-morbidities (Husain et al., 1998; Jobe, 1999; Thébaud and Abman, 2007; Islam et al., 2015; Schmidt et al., 2015). Understanding of BPD pathophysiology and prevention has evolved significantly over the past 50 years with advances in maternal care, antenatal steroids, continuous positive airway pressure (CPAP), improved ventilator strategies, surfactant therapy, and other interventions (Jobe and Bancalari, 2001; Abman et al., 2017; Owen et al., 2017). Even with these improvements in the respiratory care of preterm infants, the incidence of BPD has remained around 40% of preterm births prior to 29 weeks (Stoll et al., 2010, 2015). Further progress in reducing rates of BPD will require identification of preterm infants at greatest risk for severe disease, understanding and defining individual respiratory phenotypes and outcomes, and the ability to intervene selectively for disease prevention based on antenatal and postnatal risk factors (Lal and Ambalavanan, 2015). There is a rapidly growing story about the interactions between antenatal and early postnatal factors increasing the risk for BPD development (Lal and Ambalavanan, 2015). Data from diverse sources support the concept that BPD has its origins during fetal life and that antenatal events are potent determinants of the persistent rate of BPD despite advances in postnatal care (Taglauer et al., 2018).

Multiple insults after preterm delivery have the potential to worsen lung injury, including infections, fluid overload from a patent ductus arteriosus (PDA), mechanical ventilation, and oxygen therapy (Keszler and Sant’Anna, 2015). Mechanical ventilation is one the most life sustaining advances in neonatal care. Data from the Neonatal Research Network demonstrated that 89% of extremely low birth weight infants were treated with mechanical ventilation during the first day of life (Walsh et al., 2005). The goal of mechanical ventilation is to improve oxygenation and achieve “optimal” lung volume to minimize the adverse effects of high or low lung volumes on airways and distal lung tissue, including the developing airspace and vasculature. Appropriate ventilation settings are essential to minimize the risk for ventilator-induced lung injury (VILI) due to tissue overdistension (volutrauma; Webb and Tierney, 1974; Dreyfuss et al., 1988; Hernandez et al., 1989; Carlton et al., 1990), the cyclic collapse of bronchioles and alveoli (atelectrauma; Muscedere et al., 1994; Slutsky, 1999; Smith et al., 2013), and downstream inflammatory effects (biotrauma; Vlahakis and Hubmayr, 2005). The result of VILI is pulmonary edema, decreased lung compliance, increased cytokine production, and lung neutrophil accumulation (Patterson et al., 1988; Acute Respiratory Distress Syndrome et al., 2000; Kinsella and Abman, 2000). The development of VILI is an important determinant of the clinical course and short-term and long-term outcomes of newborns with hypoxemic respiratory failure, as well as vascular growth and the risk for pulmonary hypertension (Patterson et al., 1988; Acute Respiratory Distress Syndrome et al., 2000; Kinsella and Abman, 2000).

Previous work has demonstrated that mechanical ventilation of 1-week-old rats increases lung epithelial cell apoptosis and decreases alveolar growth (Mokres et al., 2010; Kroon et al., 2013). In addition, mechanical ventilation of infant rats acutely increases barrier disruption and cytokine release into bronchoalveolar lavage fluid (BALF; Kroon et al., 2010). These findings are in accordance with mechanical ventilation of adult rodents that is characterized by cellular damage (Hamlington et al., 2018b), inflammation (Hegeman et al., 2013), and alveolocapillary leak that leads to alveolar instability and collapse (Smith et al., 2017; Hamlington et al., 2018a). The microscale damage wrought by experimental VILI is manifested at the organ scale as increased pulmonary system elastance (reduced compliance) due to a loss of ventilated alveoli (Smith et al., 2020). The effect of alveolar instability on lung function is exacerbated at low pressures, where the damaged alveoli are more prone to collapse (Albert et al., 2009).

Inflammation is a common pathway that augments the severity of acute lung injury, which can progress to BPD (Balany and Bhandari, 2015; Leroy et al., 2018; Thebaud et al., 2019). While there are many postnatal factors that enhance lung inflammation, these exposures are often preceded by antenatal exposure to inflammation, which may further increase risk for VILI. More than 50% of pregnancies that result in very preterm births (<28 weeks gestational age) have histological evidence of chorioamnionitis, which is very often the cause of preterm labor and birth (Kim et al., 2015). Previous work from our lab has shown that a single intra-amniotic injection of endotoxin (ETX) during late gestation is sufficient to cause persistent abnormalities in lung structure and function at 2 weeks of age in infant rats (Tang et al., 2010), but whether antenatal ETX impairs lung mechanics at birth and increases severity of VILI with in the first week of life is unknown. Therefore, we hypothesize that antenatal exposure increases sensitivity to VILI in newborn rats as reflected by increased inflammation and edema along with abnormal lung mechanics. To test this hypothesis, we performed studies using an antenatal model of chorioamnionitis induced by intra-amniotic exposure to ETX and a model of VILI. The effects of these two insults were assessed shortly after birth and at an age of 1 week using detailed measurements of pulmonary mechanics, histology, alveolocapillary barrier injury, and inflammatory gene expression.

## Materials and Methods

All animal procedures were approved by The University of Colorado Denver Institutional Animal Care and Use Committee (Protocol #00339, AAALAC #00235).

### Experimental Chorioamnionitis

**Figure 1:**
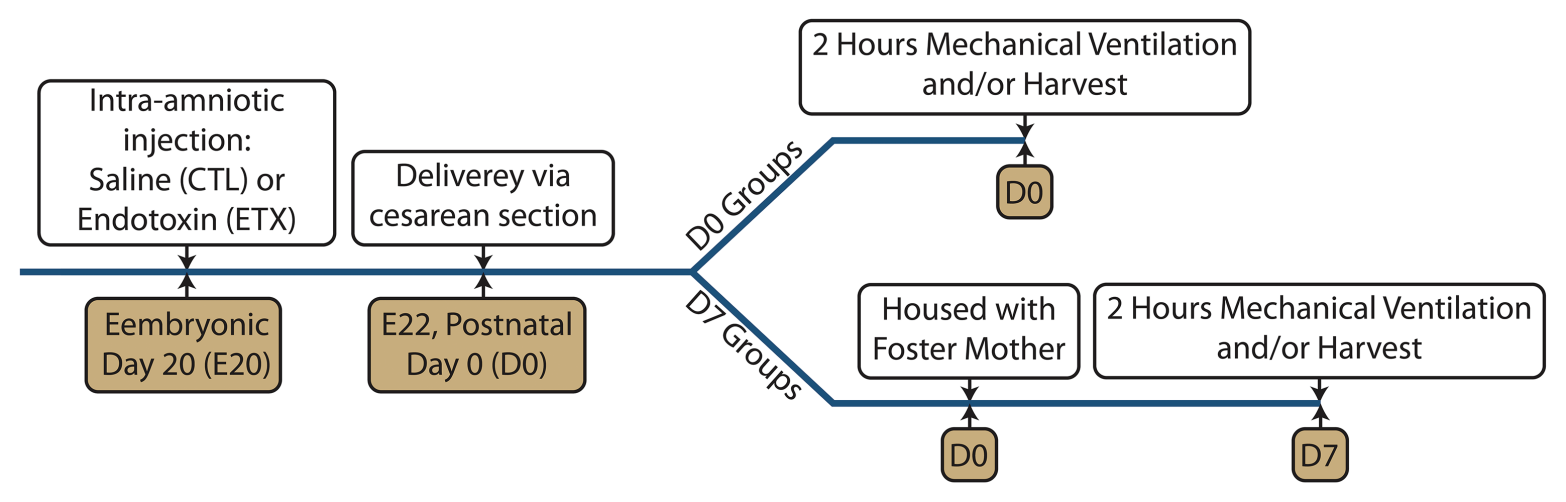
Experimental timeline showing the intra-amniotic injection at embryonic day 20 (E20), delivery *via* cesarean section at E22, and mechanical ventilation at postnatal day 0 (D0 groups) or postnatal day 7 (D7 groups).

Timed-pregnant Sprague-Dawley rats from Charles River Laboratories (Wilmington MA) were maintained in room air for at least 1 week and at embryonic day 20 (E20) received either intra-amniotic injection of saline (CTL) or 10 μg lipopolysaccharide endotoxin (ETX, List Labs #203A, Campbell CA). Pups were then delivered *via* cesarean section on E22 (Seedorf et al., 2020), which is full term. Within 30 min after birth, day 7 (D7) group pups were weighed and placed with foster mother rats in regular cages for 1 week. Day 0 (D0) group pups were weighed and ventilated within 6 h. The experimental timeline is shown in Figure 1.

**Table 1:** Experimental groups.

Antenatal injection	Postnatal group		n			Weight (g)	Mortality on ventilator (*n*)
	Age	Ventilation	Male	Female	Undetermined		
CTL	D0	NV	1	3	4	5.4 ± 0.25	N/A
CTL	D0	PROTECT	6	4	4	5.4 ± 0.41	1
CTL	D0	P20	5	3	5	5.4 ± 0.50	0
ETX	D0	NV	0	3	5	5.4 ± 0.29	N/A
ETX	D0	PROTECT	3	3	6	4.9 ± 0.55	1
ETX	D0	P20	2	2	8	5.0 ± 0.48	2
CTL	D7	NV	3	4	3	19.3 ± 2.86	N/A
CTL	D7	PROTECT	5	3	0	15.9 ± 1.53	1
CTL	D7	P20	4	4	0	16.3 ± 3.43	0
CTL	D7	P24	2	3	2	17.0 ± 2.49	0
ETX	D7	NV	2	1	7	13.2 ± 1.18	N/A
ETX	D7	PROTECT	7	1	0	14.7 ± 1.81	0
ETX	D7	P20	4	4	0	14.8 ± 0.76	0
ETX	D7	P24	4	4	1	14.7 ± 1.24	1

Antenatal treatments include saline (CTL) or endotoxin (ETX); age is 0 days (D0) or 7 days (D7); ventilation patterns are non-ventilated (NV), lung protective (PROTECT), plateau pressure of 20 (P20) or 24 (P24); *n* is the number of animals in that group. Weights are given as mean ± SD and mortality on the ventilator reports the number of animals that did not survive the full course of ventilation.

### Mechanical Ventilation

Rats were anesthetized with 5 mg/kg ketamine and 1 mg/kg xylazine *via* intraperitoneal (IP) injection in ≈50 μl (D0) or ≈150 μl (D7) saline. A tracheostomy was performed using a 24 ga, ≈19 mm plastic cannula (24Gx¾” Excel Safelet Catheter) for D0 and an 18ga, ≈10 mm metal cannula for D7. The cannula was connected to the flexiVent small animal ventilator and the baseline ventilation was started consisting of a tidal volume (Vt) = 6 ml/kg (delivered volume), positive end expiratory pressure (PEEP) = 2 cmH_2_O, respiratory rate (RR) = 250 breaths/min, and inspiratory to expiratory ratio (I:E) = 1:1.5. These settings are based on the ARDSnet-recommend Vt for lung protective ventilation (Acute Respiratory Distress Syndrome Network, 2000) and the PEEP that achieve normal blood gasses in newborn rats (Kroon et al., 2010). All ventilation was conducted with room air so that the fraction of inspired oxygen (FiO_2_) = 0.21. Body temperature was maintained with a heating pad and humidified plastic dome. Proper cannulation and the structural integrity of the respiratory system was checked by performing a recruitment maneuver (RM) consisting of a 3 s ramp to 30 cmH_2_O and a 3 s breath hold as previously described (Mandell et al., 2020). An IP injection of 0.8 mg/kg pancuronium bromide, diluted to 0.1 mg/ml in saline, was administered to prevent spontaneous breathing efforts that would invalidate the lung mechanics measurements (Mandell et al., 2020). Additional 1 mg/kg doses of ketamine were administered at 45-min intervals. This typically resulted in delivery of an additional ≈25 μl (D0) or ≈75 μl (D7) of IP saline. Heart rate was continuously monitored *via* ECG to ensure an adequate plane of anesthesia. Supplemental doses of ketamine were administered when elevated heart rate and/or animal agitation was observed. Animals were stabilized with a RM followed by 9 min of baseline ventilation interspersed with three additional RMs at 3-min intervals.

A *measurement block* was then initiated to obtain baseline lung mechanics measures. Ventilation was switched to Vt = 6 ml/kg, PEEP = 0 and a RM was performed to standardize volume history. After 10 s of ventilation, a 3 s multi-frequency (1–20.5 Hz) forced oscillation technique (FOT) measurement with onset pressure = 0, amplitude = 8 ml/kg was applied to measure pulmonary system impedance. Eight additional FOT measurements were then recorded at 16-s intervals. The constant phase model was then fit to the measured impedance (Hantos et al., 1992) to determine central airway resistance (R_N_), pulmonary system elastance (H), and tissue damping coefficient (G). The last four FOT measurements were used in the statistical analysis. Constant phase model outputs were accepted as valid if the coefficient of determination (COD) > 0.9, 0 < H < 2000 cmH_2_O/ml, and 0 < R_N_ < 5 cmH_2_O/ml/s. Another RM was then performed followed by an 11 s trapezoidal pressure-volume loop (1 s hold at 0 pressure, 3 s ramping of airway pressure from 0 up to maximum pressure of 30 cmH_2_O, 3 s hold at maximum pressure, 3 s ramp back down to 0 pressure, 1 s hold at 0). After 15 more seconds of ventilation, another RM was performed followed by a 16 s step-wise pressure-volume loop (maximum pressure of 30 cmH_2_O). A cubic spline was fit to the quasi-static points on the expiratory limb using MATLAB and the pressure-dependent compliances Cst_3_, Cst_6_, and Cst_15_ were calculated as the derivative of the cubic spline at pressures of 3, 6, and 15 cmH_2_O, respectively. Pressures of 3 and 6 cmH_2_O were selected to probe lung compliance in the range of tidal breathing and the effects of injury-induced derecruitment as we have reported for FOT measurements in mouse VILI (Hamlington et al., 2018b; Smith et al., 2020). The pressure of 15 cmH_2_O was selected because it lies in the linear portion of the upper limb of the PV loops and thus represents the behavior of the lung at high levels of inflation.

One of three ventilation patterns was then applied for 18 5-min epochs (Table 1): The PROTECT groups received the baseline ventilation, the P20 and P24 groups received a plateau pressure of 20 cmH_2_O or 24 cmH_2_O, respectively, with RR = 50 breaths/min; I:E = 1:2; and PEEP = 0 to cause VILI as we previously described in adult mice (Smith et al., 2017; Hamlington et al., 2018a,b). D0 animals did not receive P24 ventilation because of high mortality observed in pilot studies where 4/5 D0 ETX and 5/11 D0 CTL animals died on the ventilator. Around 50 μl (D0) or 150 μl (D7) saline was injected half way through the ventilation period. A second *measurement block* was recorded at the conclusion of the experiment. The NV group was not ventilated at all.

### Sample Collection and Analysis

Bronchoalveolar lavage fluid (BALF) samples were obtained at D7 by instilling and withdrawing 0.2 ml saline. Protease and phosphatase inhibitors were added to each sample, and a BCA assay was performed to measure total protein concentration. Lungs from other animals were harvested and snap frozen for biochemical analysis, or inflation fixed with 4% paraformaldehyde (20 cmH_2_O inflation pressure) and embedded in paraffin for histological analysis (Mandell et al., 2020; Seedorf et al., 2020). Total RNA was extracted from frozen lung tissue at D0 and D7 and real-time PCR was performed using Taqman probes for the housekeeping gene glyceraldehyde 3-phosphate dehydrogenase (GAPDH) and the inflammatory cytokines Interleukin (IL)-6, IL-1b, tumor necrosis factor (TNF)-a, chemokine (C-X-C motif) ligand 2 (CXCL2); and tenascin C (TEN-C) (Mandell et al., 2020).

### Verification of Animal Sex

The Extract-N-Amp (XNAT2, Sigma Aldrich) kit was used to isolate DNA from rat tail clips according to manufacturer instructions. PCR master mix was prepared using 1 μl of isolated DNA, as previously described (Dhakal and Soares, 2017). Forward primers for Kdm5c (5'-TTTGTACGACTAGGCCCCAC-3') and Kdm5d (5'-TTGGTGAGATGGCTGATTCC-3') were used with the reverse primer for Kdm5c (5'-CCGCTGCCAAATTCTTTGG-3'). A current of 110 v for 20 min was applied to the 1% agarose gel, which was subsequently imaged under UV light on the ChemiDoc XRS+ Imaging system (Bio-Rad).

### Statistical Analysis

Statistical analysis was performed with GraphPad Prism. Outliers were removed using the ROUT method (Motulsky and Brown, 2006; Q = 1%) and a Shapiro-Wilk normality test was performed. Normally distributed data were analyzed with unpaired *t*-tests or an ANOVA with a Holm-Sidak correction for multiple comparisons. Non-normally distributed data was compared with a Mann-Whitney test or a Kruskal-Wallis test with Dunn’s correction. Changes in lung function pre-ventilation to post-ventilation were assessed with paired t-tests. Data are presented as mean ± SE unless otherwise indicated. Figures show *p* < 0.05 (^*^), *p* < 0.01 (^**^), *p* < 0.001 (^***^), and *p* < 0.0001 (^****^).

## Results

### ETX Treatment Affects Lung Mechanics Prior to Prolonged Mechanical Ventilation

**Figure 2:**
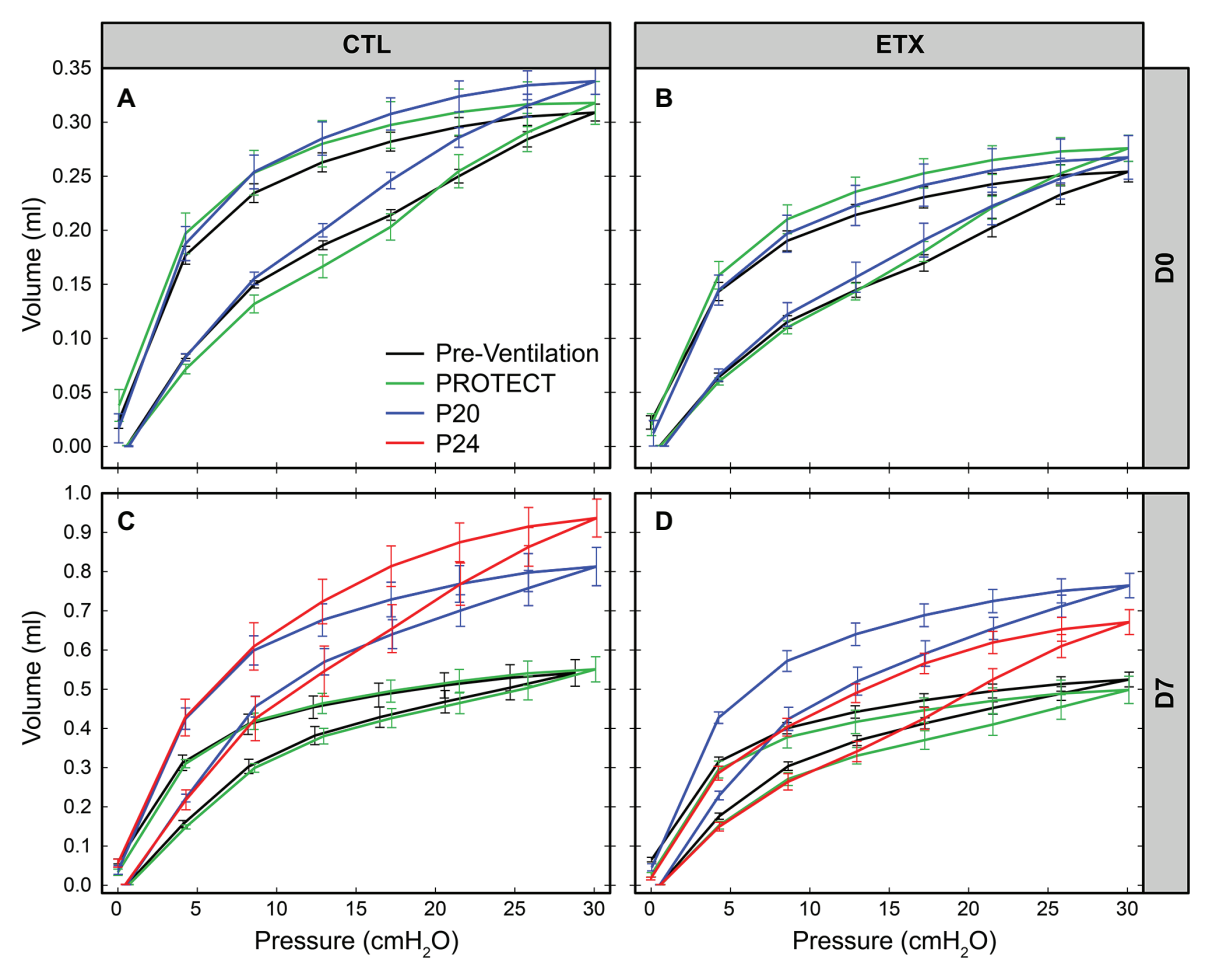
Average quasi-static pressure-volume loops for D0 (first row: **A**,**B**) and D7 (second row: **C**,**D**) for saline‐ (first column: **A**,**C**) and endotoxin-treated (second column: **B**,**D**) rats. Inspiratory capacity (IC) is reduced by ETX at D0. At D7, P20, and P24 ventilation yield an increase in IC and compliance.

**Figure 3:**
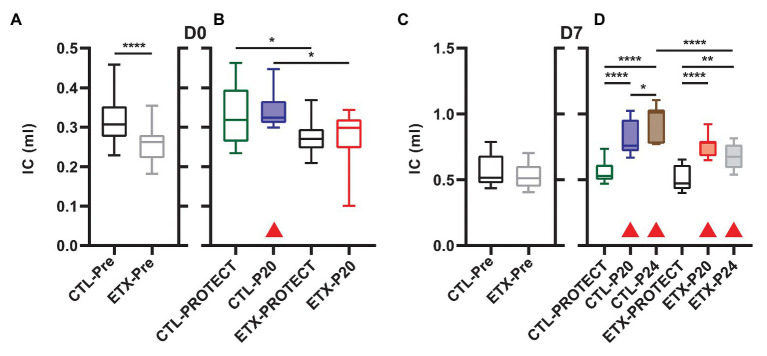
Inspiratory capacity at D0 (**A**, *n* = 23–27) and D7 (**C**, *n* = 23–25) prior to prolonged ventilation. The IC is calculated as the volume delivered during the pressure-volume loops shown in Figure 2. Ventilation at P20 resulted in increased IC in all animals except D0-ETX **(B,D)** when compared to pre-ventilation as indicated by shaded boxes and red triangles. P24 ventilation caused increased IC in both ETX and CTL at D7, and the D7 ETX treated rats have a significantly smaller IC than the CTL-P24 group.

**Figure 4:**
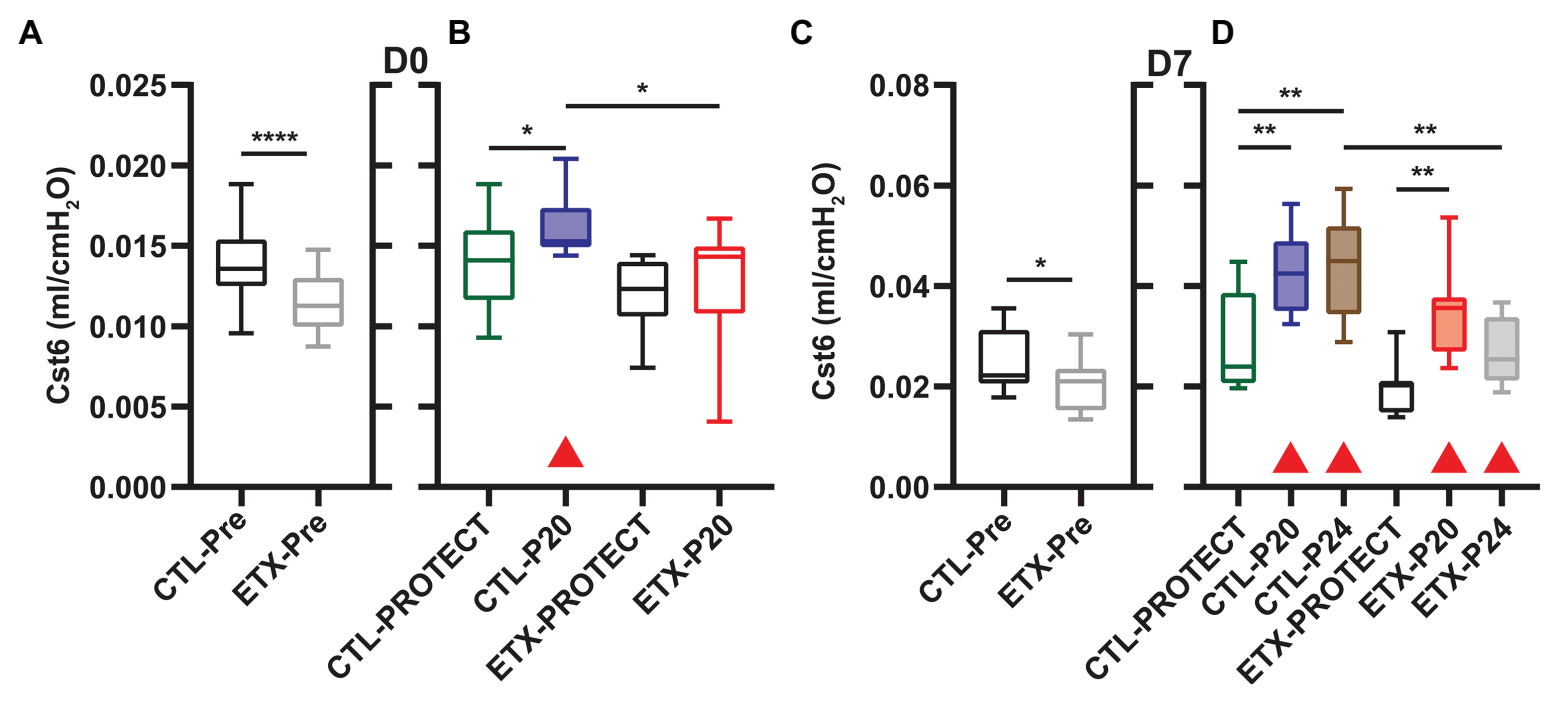
Quasi-static compliance at 6 cmH_2_O (Cst6) is representative of lung mechanics during tidal breathing for D0 **(A,B)** and D7 **(C,D)**. Prior to prolonged ventilation **(A,C)**, ETX exposure decreased Cst6 (*n* = 23–27). Cases where ventilation at P20 and P24 caused a significant increase in compliance (compared to pre-ventilation) are indicated with shaded boxes and red triangles (*n* = 7–14). Note that at D7, Cst6 was lower for ETX-P24 than CTL-P24.

**Figure 5:**
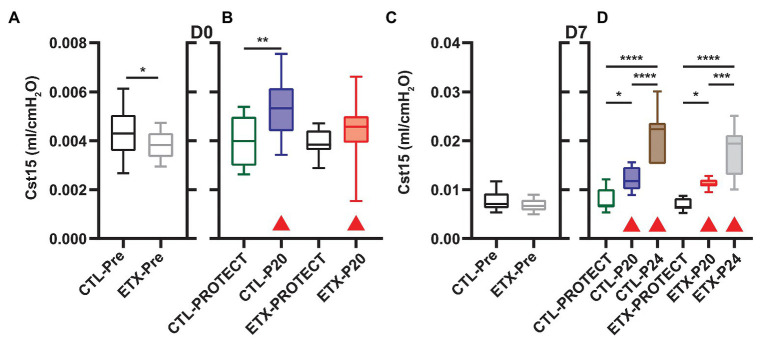
Quasi-static compliance at 15 cmH_2_O (Cst15) describes high-volume stiffness for D0 **(A,B)** and D7 **(C,D)**. Prior to prolonged ventilation **(A,C)**, ETX exposure decreased Cst15 at D0 but not D7 (*n* = 23–27). Ventilation at P20 and P24 increased Cst15 in all cases (compared to pre-ventilation) as indicated with shaded boxes and red triangles (*n* = 7–14).

The D0 ETX rats had significantly lower body weights when compared to CTL (4.91 ± 0.51 g vs. 5.43 ± 0.45 g, *p* < 0.0001), Quasi-static pressure volume loops (Figure 2) recorded before prolonged ventilation (black) show a reduction in delivered volume (IC) in D0 ETX animals (Figure 3A, *p* < 0.0001), which may reflect the smaller size of the ETX pups. However, the difference in lung volume is not solely attributed to reduced body weight since inspiratory capacity normalized by body weight (IC) was significantly greater in D0 CTL (*n* = 27) compared to D0 ETX (*n* = 23; 59.01 ± 2.34 ml/kg vs. 52.32 ± 1.63 ml/kg, *p* < 0.05). By D7, ICs were approximately equal between CTL and ETX groups (Figure 3C) even though the D7 CTL animals were significantly heavier than the D7 ETX animals. The mean and SE for the data in Figures 3–5, as well as the number of animals for each group, are provided in Supplementary Table S1.

Analysis of compliance on the expiratory limb of the quasi-static PV loops demonstrated that ETX increases pulmonary system stiffness (Figures 4, 5; Supplementary Figures S1, S4). At D0, the compliance at 6 cmH_2_O (Cst6) was significantly greater for CTL compared to ETX (Figure 4A, *p* < 0.0001). Likewise, compliance at 15 cmH_2_O (Cst15) was greater for CTL compared to ETX (Figure 5A, *p* < 0.05). These results are not unexpected given the reduced IC in D0 ETX (Figure 3A). By D7, Cst15 had normalized between CTL and ETX (Figure 5C); however, Cst6 remained lower for ETX (Figure 4C, *p* < 0.05). Taken together, these data show an ETX-induced reduction in lung volume and distensibility at birth that is nearly resolved by D7.

The age‐ and endotoxin-induced alterations in pulmonary stiffness affected delivered tidal volumes as shown in Table 2 and Supplementary Figure S5. During PROTECT ventilation, Vt trended slightly higher than the prescribed Vt = 6 ml/kg and there was a small but significant elevation in Vt when comparing D7 CTL to D0 ETX (0.63 ml/kg, *p* < 0.05). Application of P20 to the D0 animals yielded a significantly higher Vt (normalized by body weight) than in the D7 pups. It is important to note that there was no significant difference in Vt between D7-P24 and D0-P20, suggesting that those groups are subjected to comparable degrees of tissue distension. Table 2 and Supplementary Figure S5 report Vt at the end of the first ventilation epoch. Due to the ventilation-induced changes in compliance and inspiratory capacity (detailed above), tidal volumes tended to rise over the course of the ventilation, particularly in the D7-P20 and D7-P24 groups.

**Table 2:** Delivered tidal volume (ml/kg) for saline‐ (CTL) or endotoxin-exposed (ETX) pups at age 0 days (D0) or 7 days (D7).

		Tidal volume (ml/kg)		
Experimental group	Age	PROTECT	P20	P24
CTL	D0	6.5 ± 0.4	43.8 ± 6.3****	N/A
EXT	D0	6.3 ± 0.3	39.2 ± 3.9****	N/A
CTL	D7	7.1 ± 0.5*	29.2 ± 4.6	34.4 ± 4.9
EXT	D7	6.9 ± 0.3	28.2 ± 3.0	33.4 ± 2.8

Volume-controlled ventilation was set to 6 ml/kg (PROTECT), pressure-controlled ventilation was set to a plateau pressure (Pplat) = 20 cmH_2_O (P20) or Pplat = 24 cmH_2_O (P24). Symbols indicate that Vt was significantly greater than the other age cohort for that treatment/ventilation combination. *n* = 8–17 per group, table shows mean ± SD.

### PROTECT Ventilation Does Not Alter Lung Mechanics

PROTECT ventilation did not produce substantial qualitative changes in the PV loops compared to pre-ventilation loops in D0 and D7 animals (Figure 2, green). Furthermore, PROTECT ventilation did not alter IC, Cst6, and Cst15 in any of the age/treatment combinations (Figures 2, 3).

### Ventilation and ETX Treatment Synergistically Affect Quasi-Static Compliance

Ventilation at P20 and P24 caused significant increases in Cst6 and Cst15 in all age and treatment groups, when compared to pre-ventilation measurements, except for Cst6 in D0-ETX-P20 (Figure 4). Significant changes in mechanics pre‐ to post-ventilation within a group are demarcated by shaded boxes and red triangles in Figures 2, 3.

In D7 CTL, Cst6 was lower after PROTECT than P24 (Figure 4D, *p* < 0.01) and P20 (*p* < 0.01). At D7 with ETX, Cst6 was lower after PROTECT than P20 (*p* < 0.001); the D7-ETX-P24 group demonstrated lower Cst6 than the corresponding D7-CTL-P24 group (*p* < 0.01). Cst15 at D7 (Figure 5D) with CTL treatment showed progressive increases from PROTECT to P20 (*p* < 0.05) and P24 (*p* < 0.0001); the P24 group was also elevated above P20 (*p* < 0.0001). Likewise, Cst15 increased for D7-ETX from PROTECT to P20 (*p* < 0.05) and P24 (*p* < 0.0001); P24 was greater than P20 (*p* < 0.001).

At D0 with CTL treatment, Cst6 (Figure 4B) was increased for P20 in comparison to PROTECT (*p* < 0.05). This increase was not present in the ETX treated mice and, as such, Cst6 for ETX-P20 was lower than in CTL-P20 (*p* < 0.05). The D0-CTL pups had higher Cst15 after P20 ventilation than for PROTECT (*p* < 0.01).

### Ventilation and ETX Treatment Synergistically Affect Inspiratory Capacity

Inspiratory capacity (Figure 3) significantly increased pre‐ to post-ventilation with P20 (D0-CTL, D7-CTL, and D7-ETX) and P24 (D7-CTL and D7-ETX). Following ventilation at D0, IC for CTL-PROTECT was higher than ETX-PROTECT (*p* < 0.05). Likewise, IC for CTL-P20 was higher than ETX-P20 (*p* < 0.05) at D0. The D7-CTL rats showed increases in IC from PROTECT to P20 (*p* < 0.0001) and P24 (*p* < 0.0001). P24 was significantly greater than P20 (*p* < 0.05). In the D7-ETX animals, IC was lower for PROTECT than P20 (*p* < 0.0001) and P24 (*p* < 0.01). However, unlike the CTL group, ETX-P24 IC was not greater than ETX-P20 IC. As such, IC for ETX-P24 was less than CTL-P24 (*p* < 0.0001).

### Mechanical Ventilation Affects BALF Protein Concentration in D7 Rats

**Figure 6:**
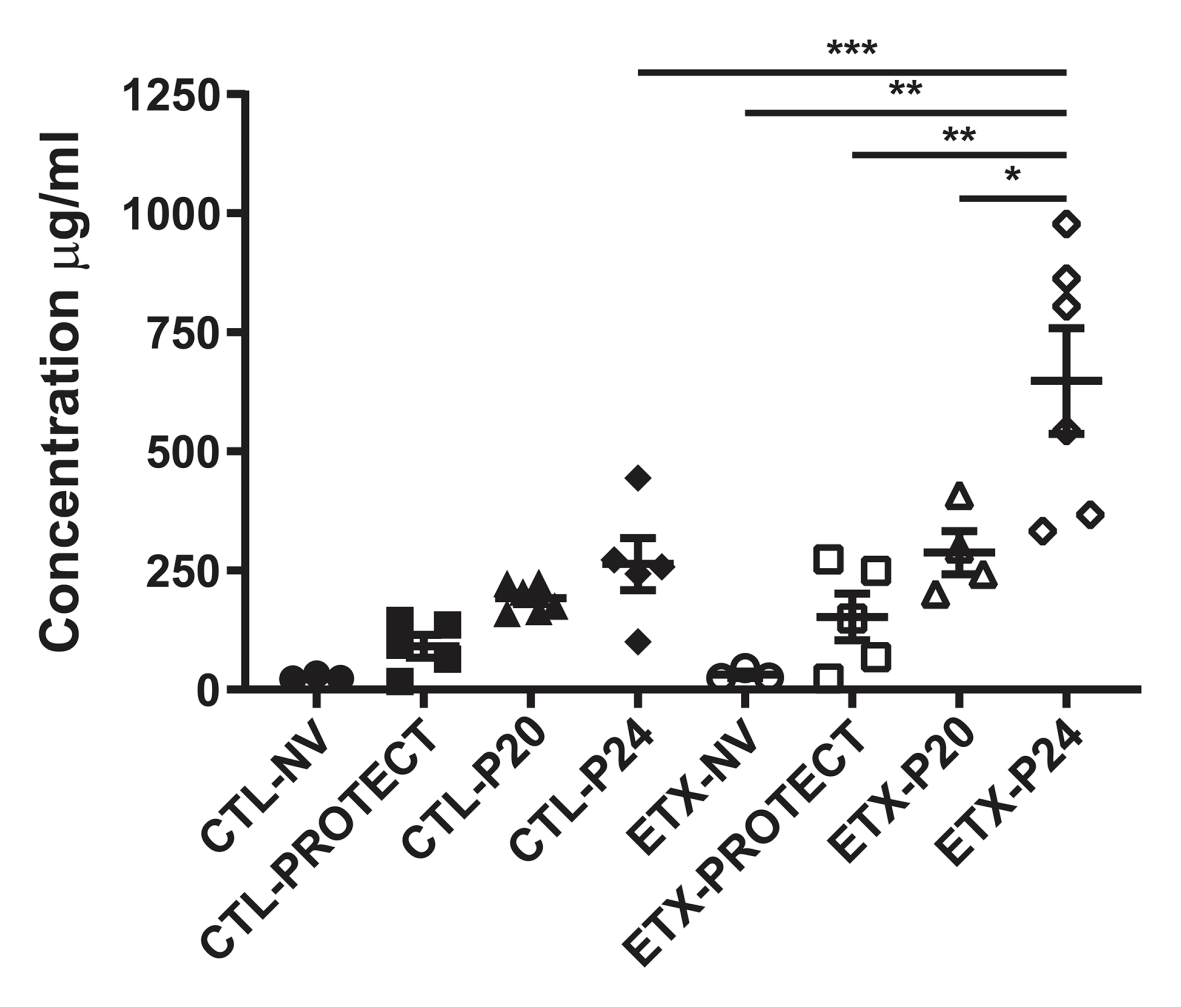
Bronchoalveolar lavage protein concentration increases with ETX and P24 ventilation treatment at day 7, where higher protein concentration suggests greater lung injury. Comparison between CTL and ETX treated rats with P24 ventilation shows that ETX-P24 treatment increases protein concentration compared to CTL-P24. Protein concentration compared among CTL shows no significance between treatment groups. Protein concentration compared among ETX treated rats demonstrates that P24 treatment increases protein concentration compared to NV, PROTECT, and P20 treatment. *N* = 3–6 per group. Error bars represent mean ± SEM.

Lung airspace protein content in BALF samples of D7 rats shows a synergy between endotoxin exposure and ventilation (Figure 6). Among the ETX groups, BALF protein concentration increased in ETX-P24 compared to non-ventilated ETX-NV (*p* < 0.01), ETX-PROTECT (*p* < 0.01), and ETX-P20 (*p* < 0.05). Among CTL groups BALF protein tended to increase with ventilation pressure, however these changes were not significant. The ETX rats ventilated with P24 showed a marked increase in BALF protein compared to CTL-P24 (*p* < 0.001). The mean, SE, and number of animals per group is provided in Supplementary Table S3.

### Mechanical Ventilation Affects Lung Structure

**Figure 7:**
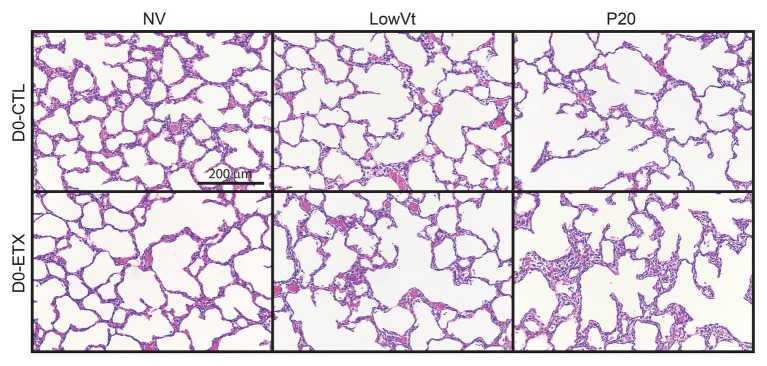
Distal lung structure at D0 demonstrates increased lung simplification and injury with ETX and mechanical ventilation. Lung histology from D0 animals (top) shows that P20 ventilation causes enlarged airspaces compared to NV and PROTECT; PROTECT ventilation demonstrates enlarged airspaces compared to NV. ETX treatment causes additional lung injury within ventilation groups. ETX-NV and ETX-PROTECT show enlarged airspace compared to CTL-NV and CTL-PROTECT, respectively.

**Figure 8:**
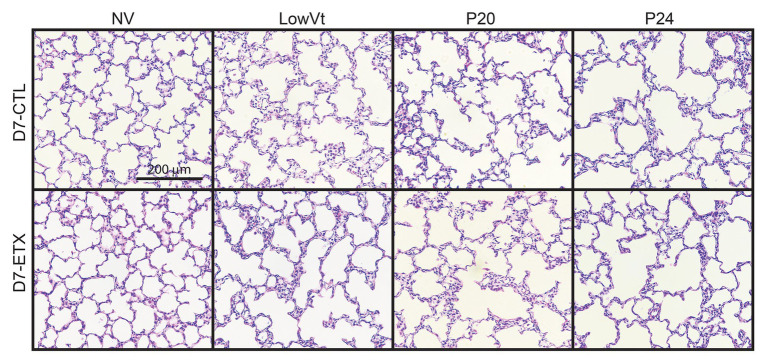
Lung histology from D7 animals demonstrates that P20 and P24 ventilation causes enlarged airspaces and loss of secondary septa compared to NV and PROTECT; PROTECT causes enlarged airspaces compared to NV. ETX treatment causes additional lung injury compared to CTL, as demonstrated by enlarged airspaces and decreased secondary septa. ETX-NV and ETX-PROTECT demonstrate a clear decrease in secondary septa compared to CTL-NV and CTL-PROTECT, respectively. ETX-PROTECT shows an increase in enlarged airspace compared to CTL-PROTECT.

Lung histology from D0 (Figure 7) and D7 (Figure 8) rats demonstrates differences in lung complexity with VILI and ETX exposure. Prior to ventilation at D0, ETX exposure caused an enlargement in parenchymal airspaces compared to CTL groups. In D7 rats, ETX exposure caused a decrease in secondary septa that is evident when comparing CTL-NV to ETX-NV. Mean linear intercepts (Supplementary Figure S7) show a significant increase with ETX treatment prior to ventilation at D7. In both D0 and D7 rats, P20 and P24 ventilation enlarged parenchymal airspaces compared to NV and PROTECT ventilation. This airspace enlargement was also visible with PROTECT ventilation, although to a lesser degree.

**Figure 9:**
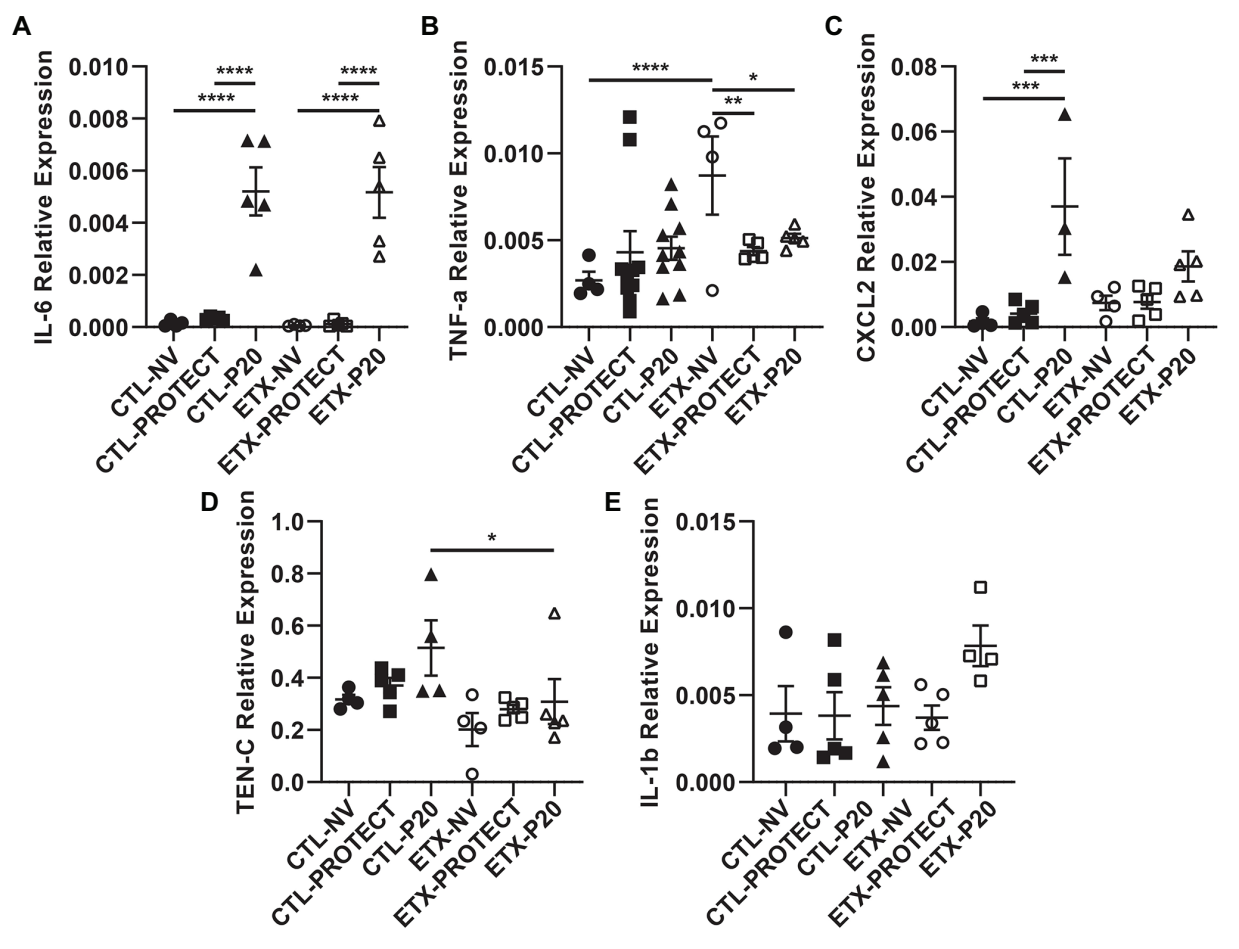
Lung tissue from D0 rats shows increases in inflammatory cytokine expression with ventilation. PCR analysis of frozen lung tissue from D0 rats demonstrates relative gene expression of inflammatory cytokines among treatment groups, where higher expression indicates increased lung inflammation. Lung tissue from D0 rats shows significantly elevated IL-6 expression after P20 ventilation compared to PROTECT and NV **(A)**, suggesting P20 treatment causes increased lung injury. Relative expression of TNF-a **(B)** and CXCL2 **(C)** trended upward with ventilation. Panels **(D)** and **(E)** show relative expression of TEN-C and IL-1b, respectively. *N* = 4–10 for TNF-a. *N* = 4–5 for all other groups. Error bars represent mean ± SEM.

### Mechanical Ventilation Affects Inflammatory Cytokine Gene Expression

Analysis of mRNA expression in whole lungs shows that higher ventilation pressures tend to increase inflammatory cytokine gene expression. In D0 animals (Figure 9), we found a striking increase in IL-6 gene expression for CTL-P20 rats compared to CTL-NV (*p* < 0.0001) and CTL-PROTECT (*p* < 0.0001). Similarly, IL-6 gene expression was markedly increased in ETX-P20 rats compared to ETX-NV (*p* < 0.0001), and ETX-PROTECT (*p* < 0.0001). TNF-a gene expression increased in ETX-NV rats compared to CTL-NV (*p* < 0.0001). Mechanical ventilation reduced TNF-a gene expression so that ETX-NV was greater than ETX-PROTECT (*p* < 0.01), and ETX-P20 (*p* < 0.05). CXCL2 gene expression increased in CTL-P20 rats compared to CTL-NV (*p* < 0.001) and CTL-PROTECT (*p* < 0.001). TEN-C gene expression increased in CTL-P20 rats compared to ETX-P20 (*p* < 0.05). We did not find any significance in IL-1b gene expression among CTL or ETX groups. The mean, SE, and number of animals in each group are provided in Supplementary Tables S4 and S5.

**Figure 10:**
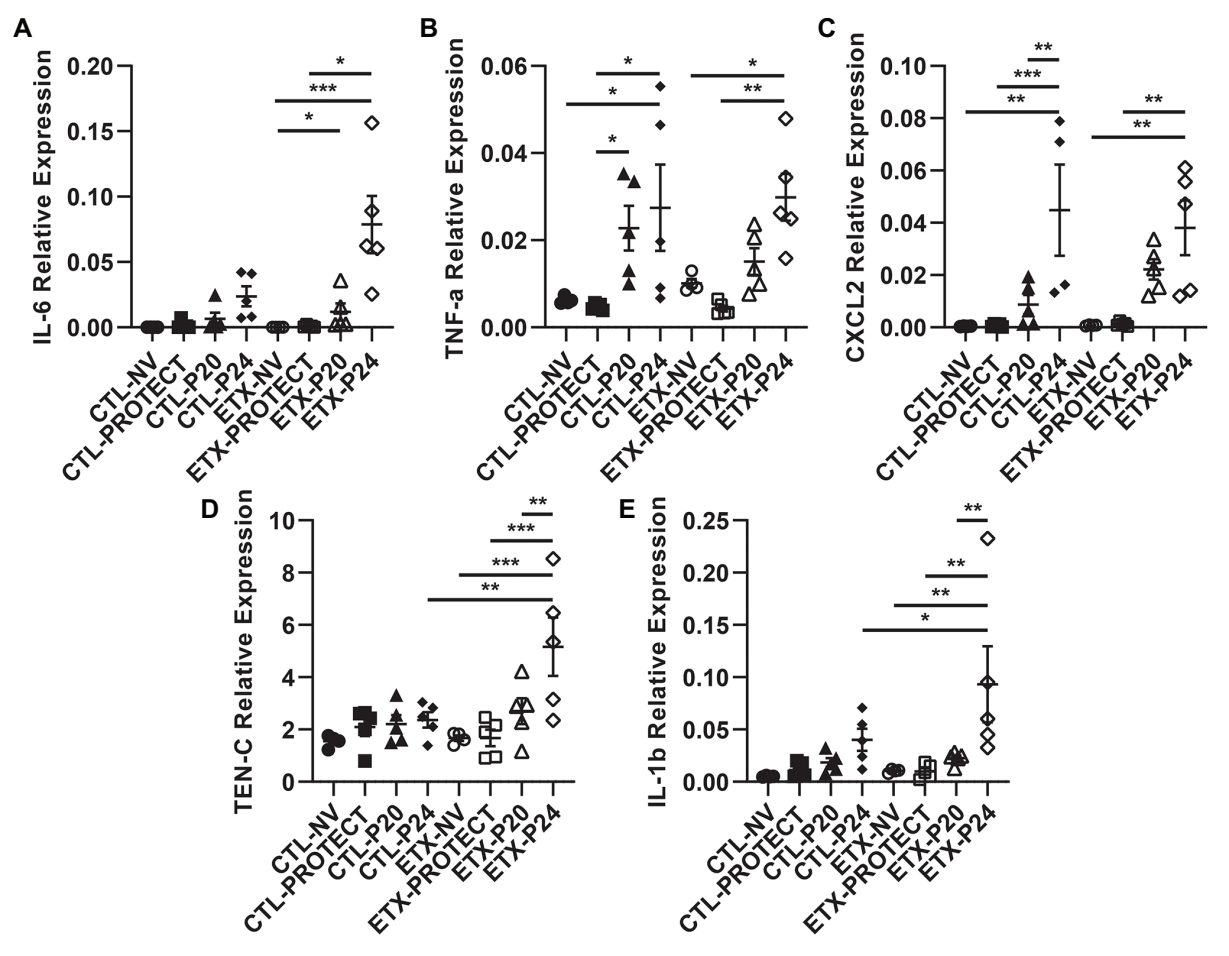
Lung tissue from D7 rats shows progressive increases in inflammatory cytokine expression with ETX and mechanical ventilation. PCR analysis of frozen lung tissue from D7 rats demonstrates relative gene expression of inflammatory cytokines among treatment groups, where higher expression indicates increased lung inflammation. Relative expression of IL-6 **(A)**, TNF-a **(B)**, CXCL2 **(C)**, TEN-C **(D)**, and IL-1b **(E)**. TEN-C and IL-1b demonstrated a significant difference between ETX-P24 treatment and CTL-P24 treatment, suggesting that ETX increases susceptibility to lung injury during VILI. *N* = 4–5 for all groups. Error bars represent mean ± SEM.

In D7 animals (Figure 10), we found that TNF-a gene expression increased in CTL-P24 compared to CTL-NV (*p* < 0.05) and CTL-PROTECT (*p* < 0.05). TNF-a gene expression also increased in CTL-P20 rats compared to CTL-PROTECT (*p* < 0.05). Likewise, TNF-a gene expression increased in ETX-P24 rats compared to ETX-NV (*p* < 0.05) and ETX-PROTECT (*p* < 0.01). CXCL2 gene expression increased in CTL-P24 rats compared to CTL-NV (*p* < 0.01), CTL-PROTECT (*p* < 0.001), and CTL-P20 (*p* < 0.01). CXCL2 gene expression increased in ETX-P24 rats compared to ETX-NV (*p* < 0.01) and ETX-PROTECT (*p* < 0.01). TEN-C gene expression increased in ETX-P24 rats compared to ETX-NV (*p* < 0.001), ETX-PROTECT (*p* < 0.001), ETX-P20 (*p* < 0.01), and CTL-P24 (*p* < 0.01). IL-1b gene expression increased in ETX-P24 rats compared to ETX-NV (*p* < 0.01), ETX-PROTECT (*p* < 0.01), ETX-P20 (*p* < 0.01), and CTL-P24 (*p* = 0.05). IL-6 gene expression increased in ETX-P24 rats compared to ETX-NV (*p* < 0.001) and ETX-PROTECT (*p* < 0.05). IL-6 expression increased in ETX-P20 rats compared to ETX-NV (*p* < 0.05). The mean, SE, and number of animals in each group are provided in Supplementary Tables S4 and S5.

Lung neutrophil content was assessed by measuring myeloperoxidase (MPO) activity in lung homogenate (Supplementary Figure S6). No significant differences were observed between groups at D0 or D7.

### Sex Differences in Lung Mechanics

We retrospectively analyzed the differences between male and female rodents for Cst3, Cst6, Cst15, and IC. With the ventilation groups pooled, there were no sex-based differences prior to prolonged ventilation. Post-ventilation, the only significant difference was found for Cst6 in the D7-CTL-PROTECT group where males (*n* = 5) showed a lower compliance than females (*n* = 3; 0.0219 ± 0.000917 vs. 0.03901 ± 0.004119, *p* < 0.05).

## Discussion

It is well known that the etiology of BPD is multifactorial and there is a complex relationship between the many antenatal and postnatal interactions that ultimately contribute to the development of BPD and late respiratory outcomes in preterm infants. Chorioamnionitis has been demonstrated to cause acute respiratory disease after birth and increases the risk for BPD (Jobe, 2012; Taglauer et al., 2018) although the pathogenic mechanisms remain unclear. Other studies have demonstrated the deleterious effects of mechanical ventilation on the preterm lung and the risk factors for abnormal lung development, BPD, and late respiratory outcomes in preterm infants (Keller et al., 2017; Morrow et al., 2017). In our current study, we show that antenatal ETX is associated with reduced inspiratory capacity at birth (Figure 3), decreased lung compliance at birth and day 7 of life (Figures 4, 5), and increased susceptibility to VILI from postnatal ventilation. Antenatal ETX rats who received high-pressure mechanical ventilation at D0 do not show increases in IC and Cst6 that are present in the saline-treated controls. At D7, antenatal ETX combined with P24 ventilation caused increased BALF protein concentration, altered lung mechanics, and increased whole-lung expression of some inflammatory mRNAs compared to saline-treated control animals receiving the same ventilation strategy. It is important to notice that even saline-treated control D0 and D7 rats who received 90 min of mechanical ventilation demonstrated increased whole-lung inflammatory transcripts and altered lung mechanics compared to non-ventilated rats. In all age and treatment groups, IC and Cst tended to increase with increasing ventilation pressure.

Preterm lungs have primitive saccules and a thickened alveolocapillary barrier at the zone of gas exchange. Infants born at 24 weeks of gestation have structurally under-developed lungs that are often unable to support gas exchange and are easily injured by mechanical ventilation due to their highly compliant chest walls and reduced lung recoil, which leave the parenchyma more susceptible to volutrauma (Schittny, 2017). At birth, rat lungs have a saccular structure, similar to preterm neonates, because alveolarization in rats occurs between postnatal days 4–21 (O’Reilly and Thebaud, 2014). Many infants born at early gestation have been exposed to chronic intra-amniotic inflammation (chorioamnionitis) that frequently leads to preterm birth (Jobe, 2012). Although these extremely preterm infants can still develop fairly normal lungs, the risk of life-long respiratory and cardiovascular impairment is high. The rats considered in the current study were exposed to an ETX model of chorioamnionitis during a phase of lung development similar to the psuedoglandular stage (24–26 weeks gestation in humans).

Although the pathogenesis of BPD is multifactorial and disease severity is modulated by the adverse effects of postnatal exposures, strong experimental and clinical data show that antenatal events are key determinants for the persistent incidence of BPD (Keller et al., 2017; Morrow et al., 2017; Taglauer et al., 2018). These clinical findings are supported by preclinical studies in which prenatal insults, even in the absence of postnatal hyperoxia or other injuries, are sufficient to cause sustained impairment of lung structure and function (Tang et al., 2010). Exposure to intrauterine inflammation results in accelerated lung maturation in the short term, but ultimately leads to the development of moderate or severe BPD, likely through initiation of the inflammatory cascade (Speer, 2009; Jobe, 2012). In experimental models, a single dose antenatal ETX exposure (delivered *via* injection into the amniotic fluid) in the absence of any postnatal injury is sufficient to stunt distal lung development with alveolar simplification, decreased pulmonary vasculature, and abnormal lung function at 2 weeks of life (Tang et al., 2010; Seedorf et al., 2020). We extend these finding to demonstrate that infant rats exposed to antenatal ETX and postnatal mechanical ventilation demonstrate increased early lung injury which, we hypothesize will worsen lung structure at 2-weeks of age. In our current study combining antenatal and postnatal exposures, we further strengthen the growing literature of antenatal exposures contributing to disease pathogenesis of BPD. Several recent prospective clinical studies have identified the impact of adverse fetal events in the pathobiology of BPD and late respiratory outcomes (Keller et al., 2017; Morrow et al., 2017). As a result, postnatal strategies to reduce the incidence and severity of BPD will likely require early identification of at risk infants with the application of early therapeutic interventions shortly after birth.

The aforementioned inflammation-induced changes in lung structure likely provide the basis for the increased susceptibility to VILI during high-pressure mechanical ventilation after antenatal ETX, as observed in the current study. Our findings show a synergy between antenatal and postnatal factors at D0 where the saline-treated control animals respond to high-pressure ventilation with increased IC, Cst6, and Cst15. In contrast, D0 rat pups exposed to antenatal ETX do not increase IC or Cst6 in response to prolonged ventilation at high inspiratory pressures, indicating an exposure-dependent early injury response to mechanical ventilation. This differential response is also seen at 7 days, where CTL pups show a progressive increase in maximal lung volumes (IC) and compliance with increasing ventilation pressure. In contrast, the ETX-P24 pups show substantially lower IC and Cst6 compared to CTL-P24 (Figure 2D). The changes are also reflected in tissue elastance (H, Supplementary Figure S1) and tissue damping (G, Supplementary Figure S2) measured with the FOT. We postulate that this proportionally lower inspiratory capacity and compliance in ETX-P24 is due to increased alveolocapillary barrier disruption, reflected in BAL protein (Figure 6), leading to alveolar flooding, derecruitment, and increased elastance (Hamlington et al., 2018a,b; Smith et al., 2020). Those pathologic features are not visible in Figures 7, 8 because the lungs were fixed *via* airway instillation, which redistributes fluid in the airspace and removes the effects of surface tension on lung structure. This fixation approach was taken due to the small size of the D0 pups. Taken together, these data indicate that lungs exposed to antenatal ETX have more rapid development of VILI in response to high-pressure mechanical ventilation.

While previous studies have demonstrated injury after mechanical ventilation for 24 h in newborn mice (Bland et al., 2007) and increased cytokine expression after prolonged mechanical ventilation (Speer, 2006), we demonstrate profound changes in lung mechanics and lung injury after just 2 h of ventilation in ETX-exposed rats at the time of birth and at the end of the first week of life. The progression of lung injury to BPD in infants is not the same as the progression that occurs in acute lung injury in adults, as the primary insults (supplemental oxygen, ventilation-mediated injury, infections, and fluid overload from PDA) in preterm infants can continuously injure the lungs, often for months, depending on the nature of respiratory support and related ventilator strategies. As such, the changes we report in the current study, after only 2 h of ventilation, highlight the importance of vigilant ventilator management in BPD infants.

The VILI-induced changes in lung mechanics, we observed for D0 and D7 rat pups are strikingly different than those we have previously reported for adult rodents (Smith et al., 2020). We predominantly observed increased inspiratory capacity and lung compliance with increasing inspiratory pressures, indicating that the lungs have become larger and more distensible. This is the opposite of what is typically observed in experimental VILI in adult rodents, where inspiratory capacity and compliance decrease with injury (Smith et al., 2020). This difference may be attributed to incomplete development of the lung and chest wall at the ages we considered. In particular, if the network of collagen fibers that supports the parenchyma and provides stiffness at high volumes to prevent overdistension is not fully developed then the lung may be “stretched out” by high-pressure ventilation. The one case where we did not observe this progressive enlargement and increased distensibility is in the ETX-P24 group. In those animals the IC, Cst3 (Supplementary Figure S4), Cst6, H, and, G indicate that ETX-P24 lungs are smaller and stiffer than CTL-P24. However, Cst15, which represents high-volume lung stiffness, is approximately equal between ETX-24 and CTL-P24, and greater than in the unventilated rats. Taken together with the increased BALF protein in ETX-P24, we postulate that both ETX and CTL lungs are “stretched” by high-pressure ventilation as reflected in Cst15. However, the ETX animals also have more alveolocapillary barrier disruption (e.g., BALF protein) leading to alveolar flooding, which reduces IC. Furthermore, the presence of proteinaceous edema causes alveoli to derecruit, and since this occurs at lower inflation pressures, the Cst3 and Cst6 are decreased while G and H are increased.

Potential limitations of our current study include use of ETX to model the effects of chorioamnionitis on fetal lung development. While this is an established model of chorioamnionitis leading to consistent findings of lung simplification similar to what is seen with human BPD, it is unlikely to reflect the complexity of chorioamnionitis in human disease. Additionally, we used short ventilation strategies with relatively high pressures and zero PEEP to induce VILI in a short time period. While these ventilation strategies are unlikely to be used in human newborns, they provide a “stress test” for the lung and thus, this model provides insight into how antenatal inflammation influences preterm lung injury from ventilation. Furthermore, other investigations have shown that long durations of ventilation at lower pressure produce similar outcomes to short ventilation durations at higher pressures (Smith et al., 2013; Wilson and Takata, 2019). Accordingly, we utilized a shorter duration at a higher pressure to reduce non-pulmonary (e.g., hemodynamic or metabolic) complications. It should also be noted that the study endpoint is to evaluate the acute lung injury in the first week of life from the combined effects of antenatal and postnatal exposures which are major risk factors for developing BPD at 2–3 months postnatal age in humans. The animals in this study are still in the alveolar stage of lung development. Future studies will address the combined effects of antenatal ETX and postnatal mechanical ventilation on long-term lung development of chronic lung disease and structural changes.

In conclusion, we demonstrate that antenatal endotoxin-exposed lungs demonstrate decreased inspiratory capacity and compliance at baseline and, when mechanically ventilated, are more susceptible to alveolocapillary barrier injury and atelectasis. The antenatal endotoxin-exposed lungs also demonstrate increased inflammatory mediators after mechanical ventilation as compared to control animals. Two hours of mechanical ventilation in saline-treated animals also significantly increased whole-lung inflammatory mediators, although to a lesser extent. This work highlights the importance of antenatal risk factors in the development of lung injury from postnatal mechanical ventilation. Increasing knowledge of the interactions of lung development, injury, and repair pathways will help determine development of effective treatments to mitigate BPD and late respiratory outcomes in the most vulnerable preterm infants.

## Data Availability Statement

The original contributions presented in the study are included in the article/Supplementary Material, further inquiries can be directed to the corresponding author.

## Ethics Statement

The animal study was reviewed and approved by University of Colorado Denver Institutional Animal Care and Use Committee.

## Author Contributions

All authors contributed to conception and design. EM, CM, GS, SR, TG, AW, EB, and BS: data collection. EM, BS, SA, TG, and GS: drafting of manuscript. EM, BS, and SA: analysis and data Interpretation. All authors contributed to the article and approved the submitted version.

### Conflict of Interest

The authors declare that the research was conducted in the absence of any commercial or financial relationships that could be construed as a potential conflict of interest.
