# Supplementary material for: Antenatal Endotoxin Impairs Lung Mechanics and Increases Sensitivity to Ventilator-Induced Lung Injury in Newborn Rat Pups
Source: Front Physiol. 2021 Jan 13;11:614283. doi: 10.3389/fphys.2020.614283 (PMC7838561; doi:10.3389/fphys.2020.614283)
Supplement: Supplementary file 1 [file Data_Sheet_1.pdf]

Antenatal Endotoxin Impairs Lung Mechanics and Increases Sensitivity to Ventilator-Induced Lung Injury in Newborn Rat Pups

Erica W. Mandell[2,4*], Courtney Mattson[1], Gregory Seedorf[2,3], Sharon Ryan[2,4], Tania Gonzalez[2,4], Alison Wallbank[1], Elisa M. Bye[2,4], Steven H. Abman[2,3], Bradford J. Smith[1,3]

[1]Department of Bioengineering, University of Colorado Denver | Anschutz Medical Campus, College of Engineering and Applied Sciences, Aurora, CO 80045.

[2]Pediatric Heart Lung Center, Department of Pediatrics, School of Medicine, University of Colorado, Aurora, CO 80045.

[3]Division of Pediatric Pulmonary and Sleep Medicine, Department of Pediatrics, School of Medicine, University of Colorado, Aurora, CO 80045.

[4]Division of Neonatology, Department of Pediatrics, School of Medicine, University of Colorado, Aurora, CO 80045.

1 Methods

MPO Assay: Total lung neutrophil activity was assessed by MPO activity. After sacrifice, lungs were removed, rinsed with PBS and snap frozen in liquid nitrogen. About 50 mg of lung tissue (1/6-1/4 of the lung) was homogenized on ice in 1 mL of ice-cold HTAB buffer (5g HTAB in 1 liter MPO buffer (0.5% HTAB). Lung homogenates were then sonicated on ice for 10 seconds with an ultrasonic homogenizer (model VC500; Sonics & Materials Inc., Danbury, CT), transferred to microcentrifuge tubes and centrifuged at 14,000 g for 15 min at 4°C. 20 µL of supernatant was transferred into a flat-bottom 96-well plate, and 200 µL of 37°C *O*-dianisidine hydrochloride solution (16.7 mg of *O*-dianisidine in 100 mL of 90% water and 10% 50mM KPO_4 buffer + 0.0005% H_2O_2 added just prior to combining with samples) was added immediately before reading the optical density at 450 nm and again 30 s later (Fisher AccuSkan FC, Fisher Scientific, Waltham, MA). MPO activity is expressed as change in optical density per minute per milligram of protein in the supernatant.

Mean Linear Intercept: The intra-alveolar and intra-alveolar duct distance was measured as the mean linear intercept (MLI) as previously described (Thurlbeck, 1967a;b). We determined MLI using an automated image processing in MATLAB version 2020b (Mathworks, Natick MA). The MLI analysis was performed on 10 randomly selected images per animal.

Radial Alveolar Counts: Alveolarization was assessed by the RAC method of Emery and Mithal (Cooney and Thurlbeck, 1982a;b). Respiratory bronchioles were identified as bronchioles lined by epithelium in one part of the wall. From the center of the respiratory bronchiole, a perpendicular line was dropped to the edge of the acinus connective tissues or septum or pleura, and the number of septa intersected by this line were manually counted.

2 Results

Forced Oscillation Technique (FOT) Measurements: Endotoxin increased elastance (H, Figure S1) and tissue damping (G, Figure S2) prior to ventilation at D0 and this difference did not persist at D7. Mechanical ventilation tended to increase H in comparison to pre-ventilation measurements, as indicated by shaded boxes and red arrows in Figure S1B and D. At D0, there were no significant post-ventilation differences between treatment groups. By D7, the EXT-P24 animals demonstrated a marked increase in H that was significantly higher than the other ETX animals as well as the CTL-P24 group. Tissue damping was also significantly higher at D7 in the EXT-P24 rats when compared to the other ETX ventilation groups and the CTL-P24 cohort. In the D0 rats, G was higher with ETX treatment than CTL when comparing the same ventilation pattern. In contrast, central airway resistance (R_N, Figure S3) was not affected by ETX at D0, but by D7 the ETX rats had significantly higher R_N. Ventilation at D0 caused a modest increase in R_N for the CTL-P20 group. At D7, no clear trends were present in the effects of ventilation on R_N.

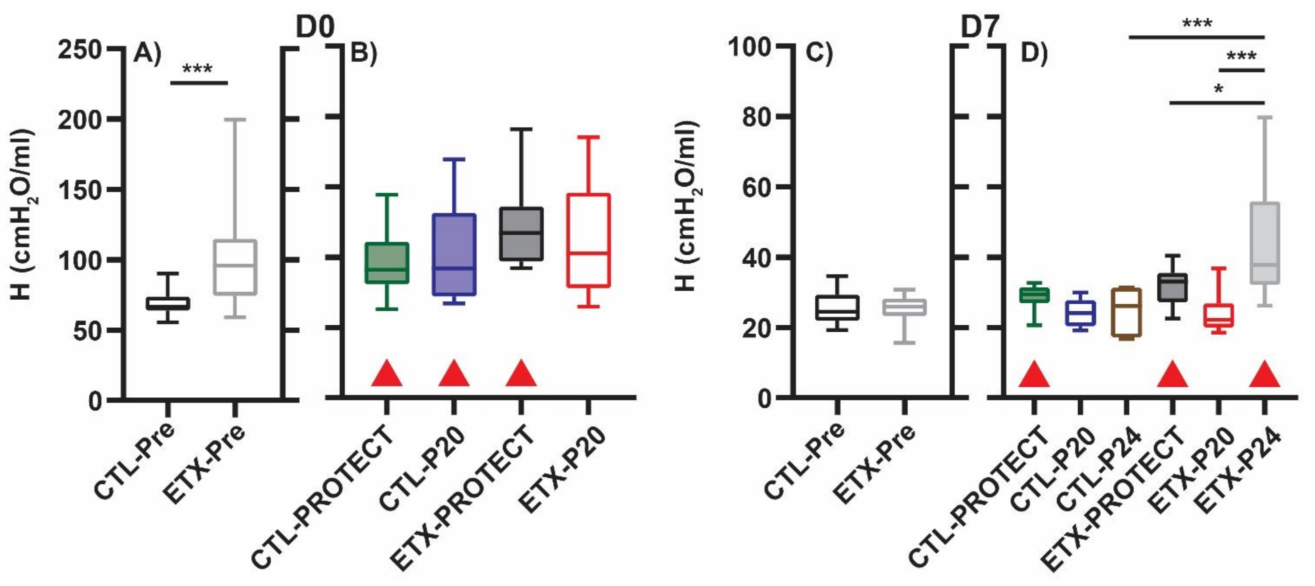

Supplementary Figure S1. Pulmonary system elastance (H) measured prior to prolonged ventilation at D0 was significantly elevated (A, n=18-25). This difference did not persist at D7 (C. n=23-25). The ETX-P24 group demonstrated increased elastance (demarcated by shaded boxes and red arrows) that was significantly greater than CTL-P24 and the other ETX ventilation groups (n=6-9).

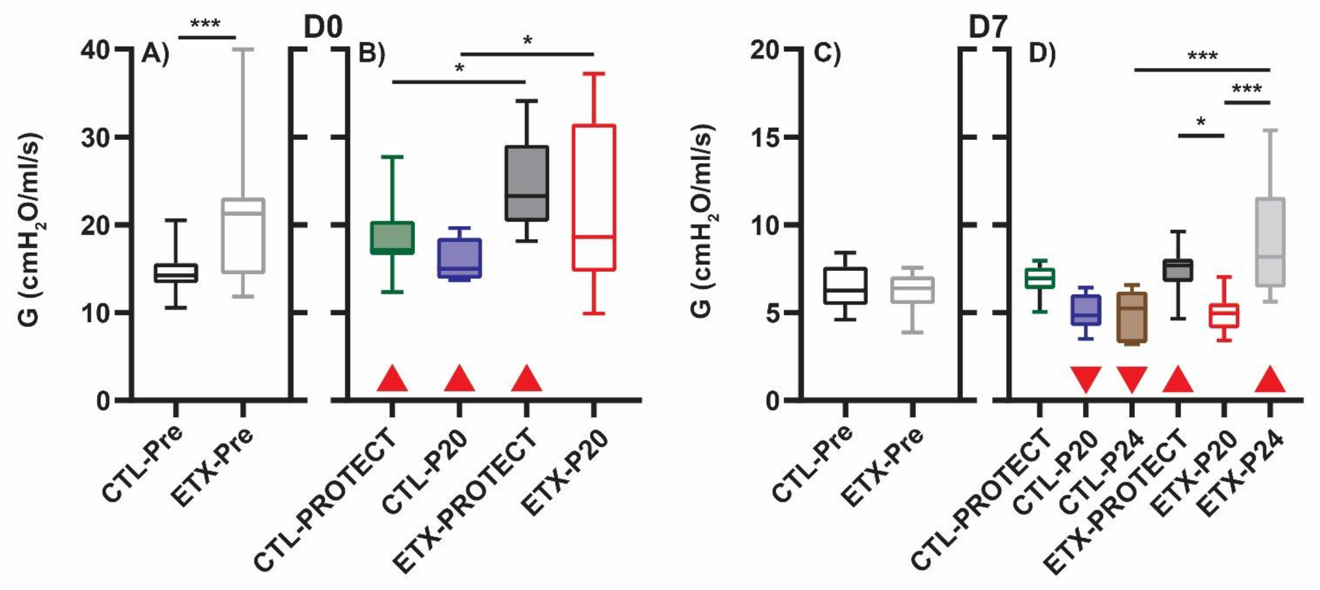

Supplementary Figure S2. Lung tissue damping (G) was significantly elevated by ETX at D0 prior to prolonged ventilation (A, n=18-25). Ventilation at D0 tended to increase G (indicated by shaded boxes and upward pointing red arrows) and significant differences were present between CTL and ETX for PROTECT and P20 (n=9-14). At D7, the CTL animals showed decreased G with P20 and P24 ventilation.

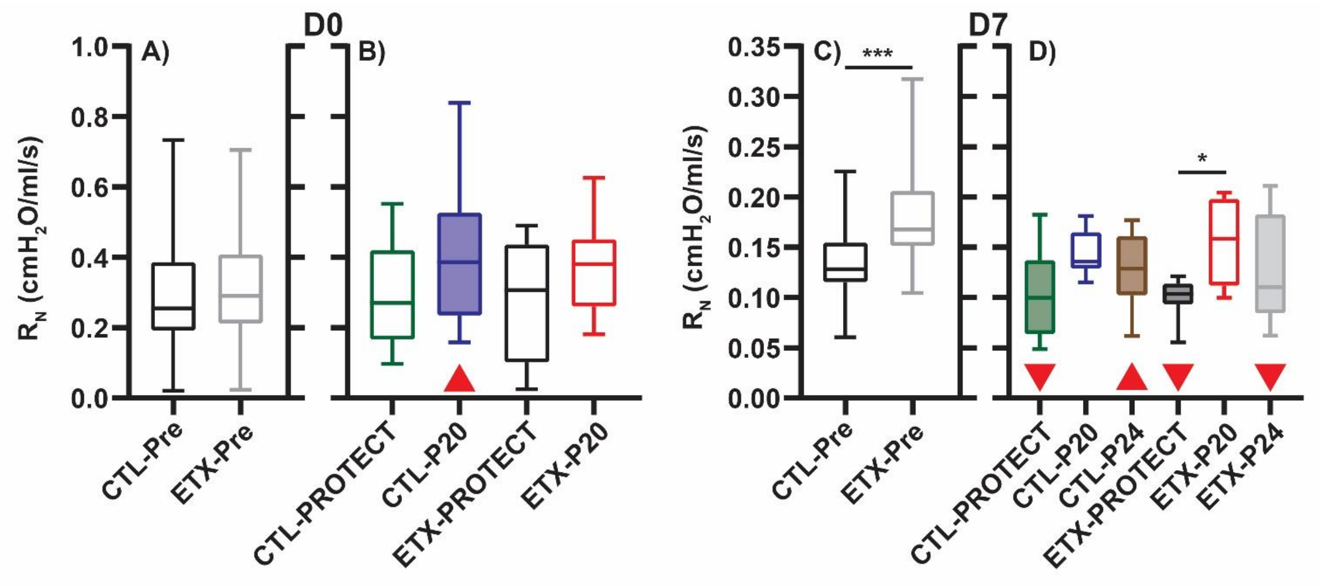

Supplementary Figure S3. Central airway resistance (R_N) was significantly elevated by ETX at D7 prior to ventilation (n=23-25). D0 did not show significant ETX effects (n=18-24). PROTECT ventilation at D7 caused a modest but significant decrease in R_N as indicated by shaded boxes and downward pointing triangles (n=8). Ventilation of CTL-P20 (D0) and CTL-P24 (D7) rats caused a modest increase in R_N that is indicated by upward pointing triangles.

Quasi-static Compliance: The quasi-static compliance calculated at an airway pressure of 3 cmH_2O (Cst3) followed the same trends as Cst6 (Figure S4). Prior to ventilation, D0 ETX animals demonstrated a marked decrease in lung compliance that did not persist at D7. Ventilation of the D0 pups caused increased compliance in all but the CTL-PROTECT group, and the CTL-P20 animals showed higher Cst3 than ETX-P20. At D7, increasing ventilation pressure tended to increase Cst3 with the exception of ETX-P24.

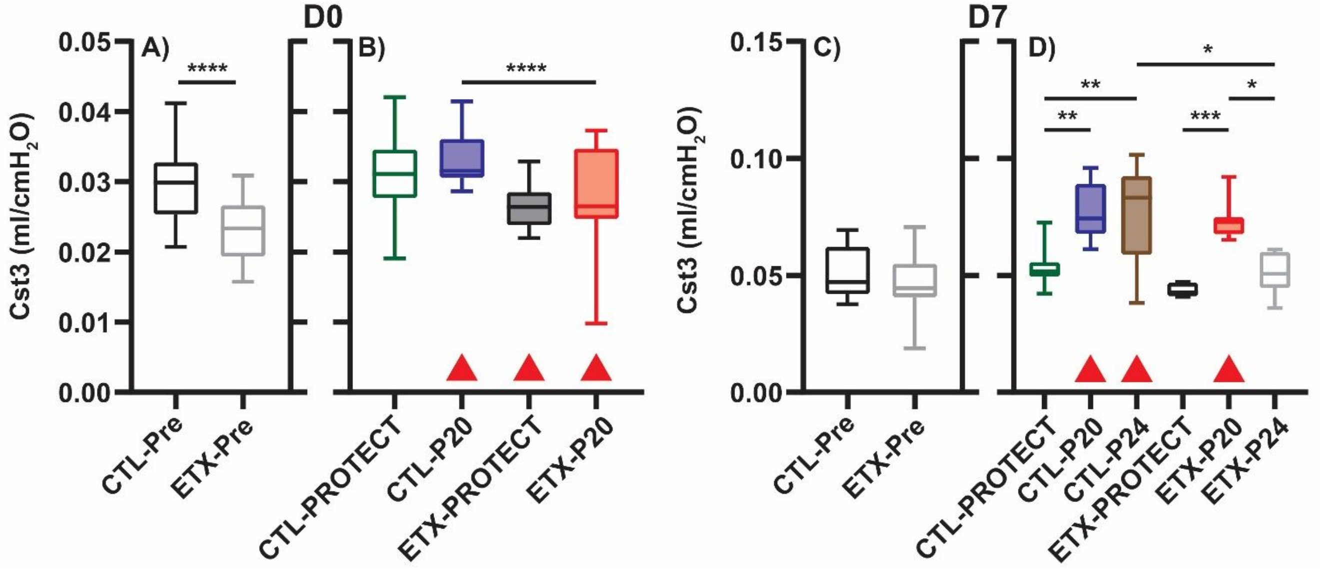

Supplementary Figure S4. Pulmonary system compliance at a pressure of 3 cmH_2O (Cst3) is significantly decreased by ETX at D0 prior to prolonged ventilation (A, n=23-27). Ventilation tended to increase Cst3 (indicated by shaded boxes and red triangles). P24 ventilation at D7 increased Cst3 in CTL but not in ETX.

Tidal Volume: The delivered tidal volume (scaled by body weight) was calculated from ventilator waveform data that was corrected for cannula resistance and gas compressibility by the ventilator software. During PROTECT ventilation (Figure S5A) Vt was elevated slightly above the prescribed 6 mL/kg and the D7-CTL animals showed significantly higher PROTECT Vt than D0-CTL. During P20 ventilation (Figure S5B, blue) the D0 animals showed significantly higher Vt than the D7 animals. However, D0-P20 and D7-P24 tidal volumes were similar, suggesting that those two groups may be compared directly.

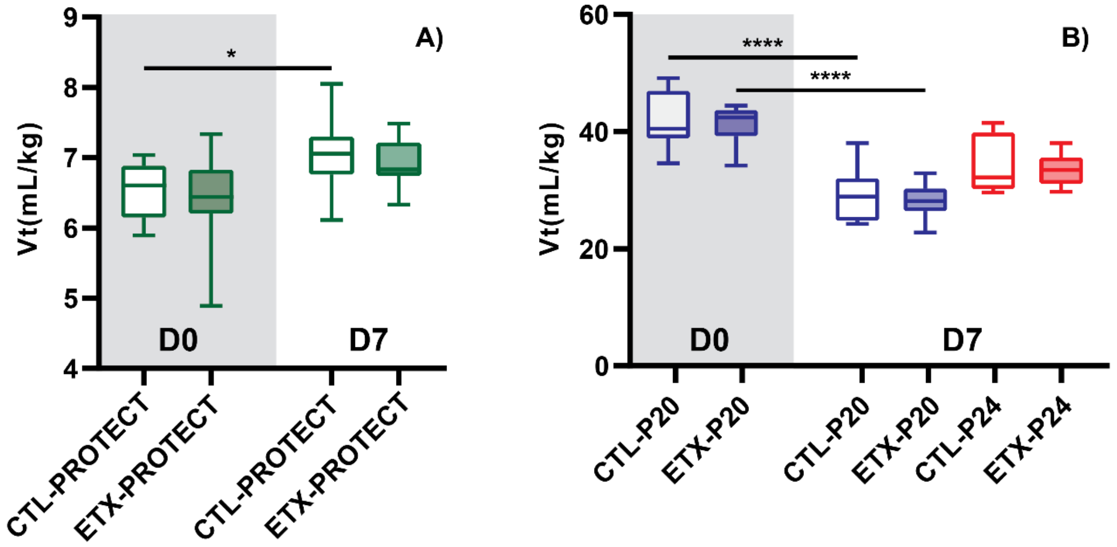

Supplementary Figure S5. Delivered tidal volume for D0 (grey shaded) and D7 pups during PROTECT (green, n = 11-17), P20 (blue, n = 8-14), and P24 ventilation (red, n = 8). Shaded boxes indicate endotoxin treatment (ETX).

Lung Neutrophil Content: Myeloperoxidase (MPO) activity in lung homogenate was measured to assess lung neutrophil content (Figure S6) and no significant differences were observed. At D0, there was a trend towards increased MPO activity in the lungs of animals exposed to antenatal ETX prior to postnatal ventilation. At D7, there was trend towards increased MPO activity with increasing ventilation pressure from PROTECT to P20 to P24.

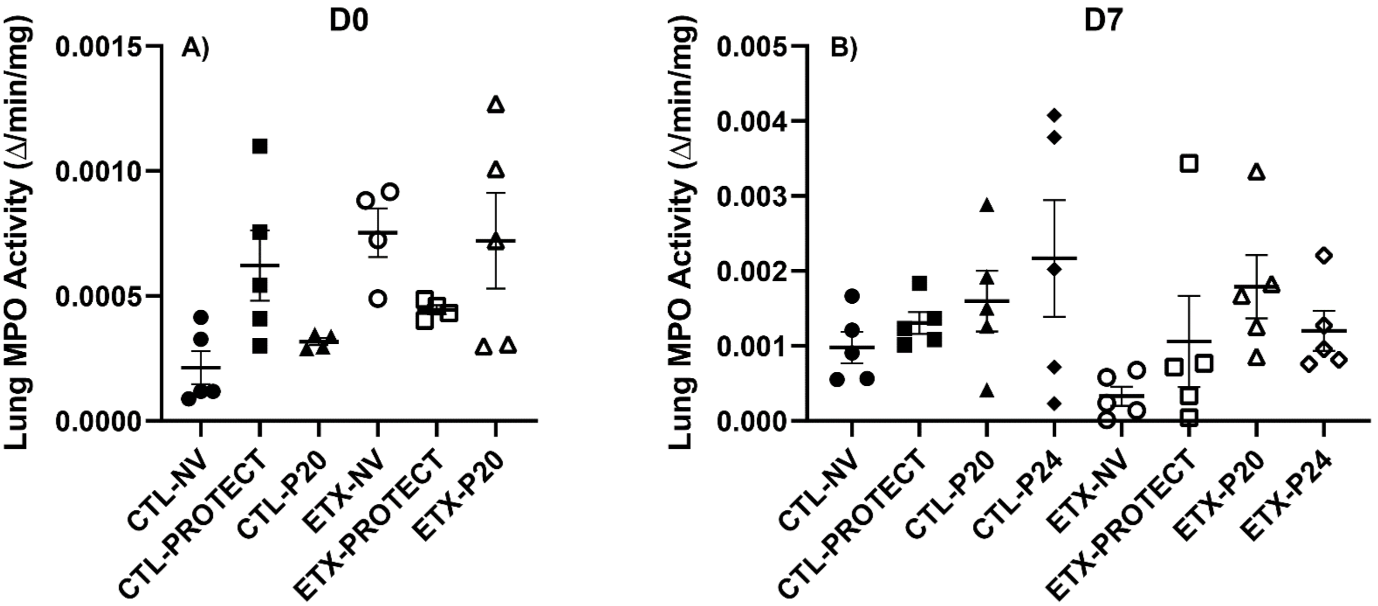

Supplementary Figure S6. Measurement of Myeloperoxidase (MPO) activity in whole lung tissue of animals at birth (D0) and day 7 of postnatal life (D7). There was a not a significant change in MPO activity in animals exposed to antenatal endotoxin (ETX) and postnatal mechanical ventilation as compared to animals who received postnatal mechanical ventilation alone at D0 or D7 (n=4-5).

Lung Structure: At D7 prior to ventilation, ETX increased the mean linear intercept (MLI, Figure S7) and there was a trend towards decreased radial alveolar counts (RAC, Figure S8). Both of these findings point towards alveolar simplification with ETX treatment. Mechanical ventilation of D7-ETX caused a *decrease* in the MLI which, given that total lung volumes tended to increase, was an unexpected finding. It is possible that this observation stems from differential structural changes in the alveoli and alveoli ducts. Figure 8 shows substantial enlargement of the alveolar ducts due to the retraction of the alveolar septa. Our prior studies in mouse ventilator induced lung injury demonstrate a reduction in alveolar size while volume is shifted to the ducts (Smith et al., 2020). In that case, the MLI could conceivably decrease as there are many more alveoli than ducts in the numeric average. Technical aspects of the tissue preparation, such as variable lung inflation levels, could also explain contribute to this somewhat unexpected result. At D0, no significant changes were observed in the MLIs.

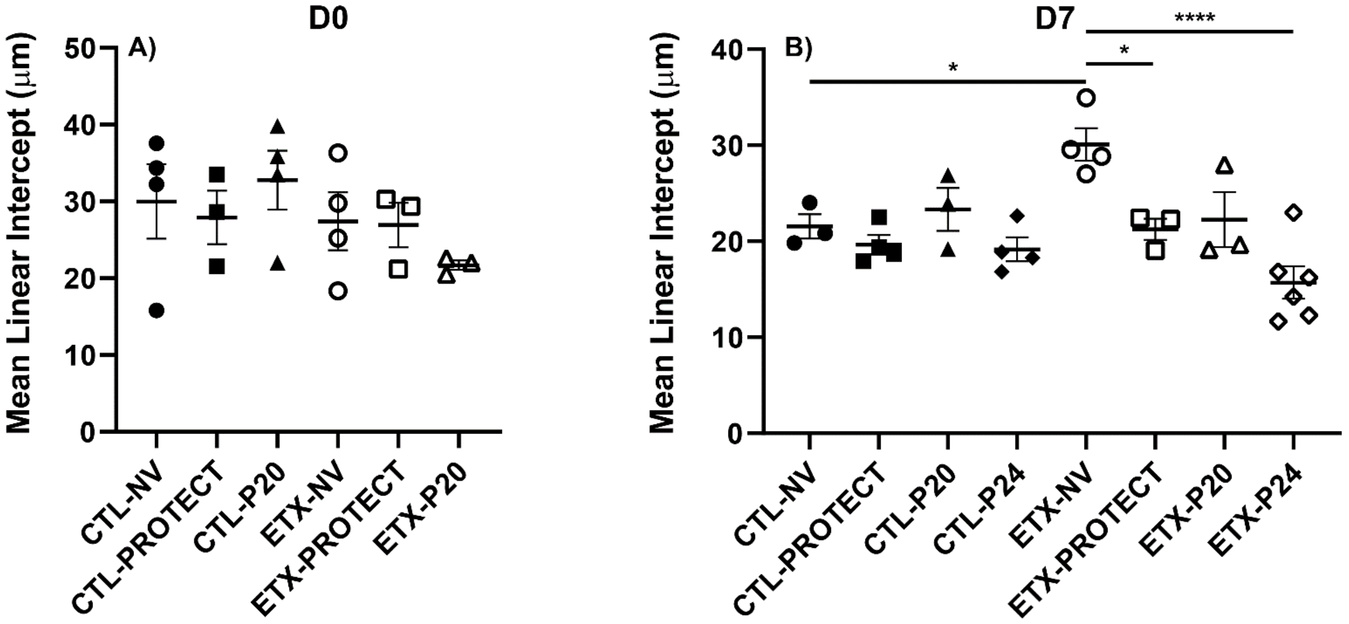

Supplementary Figure S7. Mean linear intercept (MLI) prior to mechanical ventilation (NV) was increased by antenatal endotoxin (ETX) at day 7 of life (D7). In the D7-ETX pups, mechanical ventilation (PROTECT and P24) decreased the MLI. At D0, no significant alterations were observed. (n=3-6).

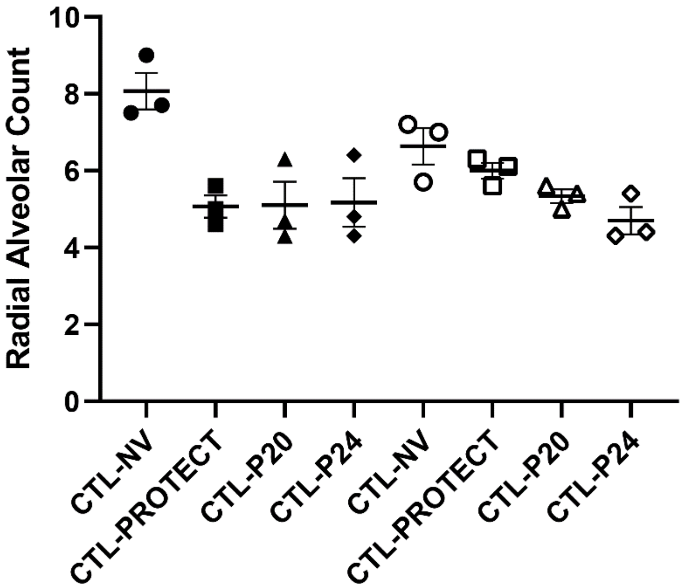

Supplementary Figure S8. Radial alveolar counts (RACs) at day 7 of life (D7) are a measure of lung complexity. Prior to mechanical ventilation, antenatal endotoxin treatment (ETX) tended to decrease the RAC. Mechanical ventilation tended to decrease RAC in both ETX and control (CTL) pups. n=3.

Supplementary Tables: The supplementary tables show the number of animals (n), the mean, and the standard error (SE). Table S1 shows lung mechanics derived from the quasi-static pressure volume loops (IC, Cst3, Cst6, and Cst15). Table S2 summarizes the results of the FOT measurements. Table S3 details the BAL protein measurements in the D7 rats, and Tables S4 and S5 show gene expression for both D0 and D7.

			IC (ml)		Cst3 (ml/cmH$_2$O)		Cst6 (ml/cmH$_2$O)		Cst15 (ml/cmH$_2$O)	
		N	Mean	SE	Mean	SE	Mean	SE	Mean	SE
D0	CTL-NV	27	0.317	0.0104	0.0294	0.00090	0.0141	0.00043	0.0043	0.00017
	CTL-PROTECT	14	0.328	0.0194	0.0308	0.00162	0.0140	0.00081	0.0041	0.00027
	CTL-P20	13	0.344	0.0125	0.0333	0.00103	0.0162	0.00048	0.0053	0.00031
	ETX-NV	23	0.257	0.0092	0.0233	0.00093	0.0113	0.00035	0.0038	0.00011
	ETX-PROTECT	11	0.274	0.0125	0.0264	0.00107	0.0121	0.00065	0.0039	0.00015
	ETX-P20	12	0.278	0.0190	0.0277	0.00215	0.0128	0.00099	0.0044	0.00034
D7	CTL-NV	23	0.577	0.0248	0.0517	0.00220	0.0253	0.00120	0.0076	0.00037
	CTL-PROTECT	8	0.562	0.0308	0.0535	0.00353	0.0284	0.00344	0.0079	0.00083
	CTL-P20	8	0.819	0.0481	0.0773	0.00434	0.0428	0.00287	0.0121	0.00090
	CTL-P24	7	0.940	0.0519	0.0742	0.00834	0.0433	0.00401	0.0211	0.00202
	ETX-NV	25	0.527	0.0188	0.0470	0.00225	0.0206	0.00097	0.0068	0.00025
	ETX-PROTECT	8	0.501	0.0350	0.0435	0.00112	0.0198	0.00189	0.0068	0.00044
	ETX-P20	8	0.768	0.0310	0.0737	0.00292	0.0351	0.00326	0.0112	0.00036
	ETX-P24	9	0.674	0.0320	0.0513	0.00297	0.0275	0.00222	0.0177	0.00164

Supplementary Table S1: Inspiratory capacity (IC) and the quasi-static compliance at 3 (Cst3), 6 (Cst6), and 15 cmH$_2$O (Cst15). n is the number of animals in the analysis, SE is the standard error.

		n	H (cmH_2O/ml) Mean	H SE	G ($cmH_2O/ml/s$) Mean	G SE	R_N ($cmH_2O/ml/s$) Mean	R_N SE
D0	CTL-NV	25	68.89	1.72	14.62	0.45	0.29	0.033
	CTL-PROTECT	14	95.95	6.15	18.46	0.99	0.29	0.039
	CTL-P20	12	103.10	10.55	15.95	0.76	0.39	0.057
	ETX-NV	18	102.60	8.81	21.65	1.82	0.32	0.042
	ETX-PROTECT	9	122.30	10.21	24.66	1.84	0.27	0.058
	ETX-P20	10	110.00	12.44	21.82	2.97	0.38	0.043
D7	CTL-NV	23	25.51	0.94	6.45	0.25	0.13	0.008
	CTL-PROTECT	8	28.59	1.35	6.85	0.33	0.10	0.016
	CTL-P20	8	24.19	1.37	4.98	0.36	0.14	0.008
	CTL-P24	6	24.85	2.67	4.94	0.58	0.13	0.016
	ETX-NV	25	25.27	0.77	6.14	0.21	0.18	0.010
	ETX-PROTECT	8	31.97	2.01	7.48	0.51	0.10	0.007
	ETX-P20	8	24.04	2.08	4.97	0.40	0.16	0.016
	ETX-P24	9	44.12	5.91	9.00	1.14	0.13	0.018

Supplementary Table S2: Pulmonary system elastance (H), tissue damping (G), and central airways resistance (R_N) measured with the forced oscillation technique. n is the number of animals in the analysis, SE is the standard error.

		n	Mean (μg/mL)	SE
D7	CTL-NV	3	25.94	3.266
	CTL-PROTECT	5	90.98	23.63
	CTL-P20	6	191.8	11.91
	CTL-P24	5	263.6	54.55
	ETX-NV	3	31.57	6.677
	ETX-PROTECT	5	152.4	48.86
	ETX-P20	4	287.5	44.84
	ETX-P24	6	648.2	110.9

Supplementary Table S3: Protein concentration of BALF samples of D7 rats. n is the number of animals in the analysis, SE is the standard error.

		IL-6			TNF-a			CXCL2		
		n	Mean	SE	n	Mean	SE	n	Mean	SE
D0	CTL-NV	4	0.00014	5.79E-05	4	0.00269	0.00050	4	0.00170	0.00100
	CTL-PROTECT	5	0.00033	2.97E-05	10	0.00431	0.00122	5	0.00415	0.00142
	CTL-P20	5	0.00521	0.00092	10	0.00454	0.00066	3	0.03700	0.01482
	ETX-NV	4	6.27E-05	1.45E-05	4	0.00873	0.00224	4	0.00745	0.00224
	ETX-PROTECT	5	0.00012	4.98E-05	5	0.00437	0.00023	5	0.00773	0.00212
	ETX-P20	5	0.00516	0.00097	5	0.00512	0.00024	5	0.01862	0.00460
D7	CTL-NV	4	7.05E-05	1.77E-05	4	0.00621	0.00042	4	0.00039	6.35E-05
	CTL-PROTECT	5	0.00186	0.00136	5	0.00484	0.00033	5	0.00071	0.00026
	CTL-P20	5	0.00653	0.00460	5	0.02276	0.00514	5	0.00872	0.00366
	CTL-P24	5	0.02370	0.00764	5	0.02744	0.00991	4	0.04482	0.01742
	ETX-NV	4	7.28E-05	9.87E-06	4	0.01011	0.00098	4	0.00078	9.53E-05
	ETX-PROTECT	5	0.00085	0.00039	5	0.00432	0.00066	5	0.00126	0.00036
	ETX-P20	5	0.01181	0.00654	5	0.01507	0.00307	5	0.02216	0.00390
	ETX-P24	5	0.07869	0.02188	5	0.02985	0.00539	5	0.03801	0.01045

Supplementary Table S4: Relative expression of inflammatory cytokines IL-6, TNF-a, and CXCL2 in whole-lung tissue of D0 and D7 rats. n is the number of animals in the analysis, SE is the standard error.

3 References:

Cooney, T.P., and Thurlbeck, W.M. (1982a). The radial alveolar count method of Emery and Mithal: a reappraisal 1--postnatal lung growth. *Thorax* 37**,** 572-579.

Cooney, T.P., and Thurlbeck, W.M. (1982b). The radial alveolar count method of Emery and Mithal: a reappraisal 2--intrauterine and early postnatal lung growth. *Thorax* 37**,** 580-583.

Smith, B.J., Roy, G.S., Cleveland, A., Mattson, C., Okamura, K., Charlebois, C.M., Hamlington, K.L., Novotny, M.V., Knudsen, L., Ochs, M., Hite, R.D., and Bates, J.H.T. (2020). Three Alveolar Phenotypes Govern Lung Function in Murine Ventilator-Induced Lung Injury. *Frontiers in Physiology* 11.

Thurlbeck, W.M. (1967a). The internal surface area of nonemphysematous lungs. *Am Rev Respir Dis* 95**,** 765-773.

Thurlbeck, W.M. (1967b). Measurement of pulmonary emphysema. *Am Rev Respir Dis* 95**,** 752-764.
